# Global profile of anemia during pregnancy versus country income overview: 19 years estimative (2000–2019)

**DOI:** 10.1007/s00277-023-05279-2

**Published:** 2023-05-26

**Authors:** Evelyn Araujo Costa, Jackline de Paula Ayres-Silva

**Affiliations:** 1 Distance Education Center of Rio de Janeiro State (CEDERJ), Rio de Janeiro, Brazil; 2 Laboratory of Pathology, Oswaldo Cruz Institute - Oswaldo Cruz Foundation (Fiocruz), Rio de Janeiro, Brazil

**Keywords:** Anemia, Iron deficiency, Pregnant women, Low-middle income countries, Africa

## Abstract

Anemia is a worldwide public health problem that is worst in low- and middle-income countries (LMICs), reaching 60% of prevalence. The etiology of anemia is diverse and multifactorial, with iron deficiency being the most prevalent, and often found in pregnant women. Iron is indispensable for the production of red blood cells and approximately 80% of the available heme iron is used for hemoglobin synthesis in mature erythroblasts. Iron deficiency affects oxygen transport, compromising energy and muscle metabolism and can occur with depletion of iron storage, defective erythropoiesis, and low hemoglobin levels. We analyzed anemia prevalence in pregnant women from 2000 to 2019 worldwide correlating them with current (2022) country income, with especial attention to LMICs using WHO dataset. Our analysis indicates that pregnant women from LMICs had a higher probability (40%) of anemia during pregnancy especially those from Africa and South Asia. Africa and the Americas showed a higher decrease in the prevalence of anemia from 2000 to 2019. The Americas and Europe have a lower prevalence, concentrated in 57% of most upper-middle- and high-income countries. Black women are also more prone to develop anemia during pregnancy, especially if they are from LMICs. However, the prevalence of anemia appears to decrease with an increase in educational level. In conclusion, anemia prevalence fluctuated from 5.2 to 65.7% worldwide in 2019, validating it as a public health problem.

**Supplementary Information:**

The online version contains supplementary material available at 10.1007/s00277-023-05279-2.

## Introduction

Anemia is a worldwide public health problem, in which one-third of reproductive women, 38% of pregnant adult females, and children under 5 years old are anemic, being worst in low- and middle-income countries (LMICs) [1–4]. The etiology of anemia is diverse and multifactorial, with iron deficiency being the most prevalent. Groups with high metabolic demands, such as pregnancy, lactation, and children, are the most affected, with many symptoms associated with impaired oxygen delivery to tissues, such as motor developmental problems, mental fatigue, and difficulty concentrating. In pregnant women, anemia can increase the risk associated with low birth weight, preterm labor, and perinatal mortality [1].

Anemia is a hematological condition in which hemoglobin (Hb) concentrations are under predetermined ranges defined by a large and heterogeneous population worldwide [4, 5]. For women, Hb reference ranges from 12 to 16 g/dL, being < 120 g/L for non-pregnant and < 110 g/L for pregnant women. Currently, there are proposals to review these ranges to adjust to population needs, such as ethnicity, age, and sex [6], and one of the first efforts were National Health and Nutrition Examination Survey – III (NHANES-II) and Scripps-Kaiser database [7].

In LMICs, the prevalence of anemia is principally caused by food shortages, in contrast to developed countries, where hemoglobinopathies such as thalassemia and sickle cell anemia represent a large part of the statistics [1, 8], but also reported in LMICs in sub-Saharan Africa and Middle East [9]. Anemia can have different origins, and adequate investigation is required to start treatment as soon as possible. Among the existing possibilities are hereditary blood disorders such as hemoglobinopathies, parasitic diseases such as hookworm and schistosomiasis, malaria infections, and deficiencies of folate, iron, and B vitamins. 

Iron deficiency is the most frequent cause of anemia, and often found in pregnant women. Iron deficiency usually leads to decreased hemoglobin levels and red blood cell counts, which reflects in lower hematocrit and hemoglobin concentrations. However, there are many other causes of anemia not related to iron deficiency [5], as well as iron deficiency without anemia [10]. Pregnant women require higher concentrations of nutrients to meet the physiological demands during the gestational period. Data corresponding to anemia in pregnancy can range from a low rate in countries with a high income, such as the USA (16%), to as high as 63% in Yemen, a low-income country. This large variation worsens among minorities in the economic, social, and ethnic fields [11, 12]. Globally, this rate is 40%, and in Brazil, it is 37% according to the WHO [4, 13]. The prevalence of anemia among Brazilian adults and the elderly is approximately 9.9%, but more severe cases are seen among women, the elderly, people with low schooling, Blacks, and residents of the North and Northeast regions, with normocytic normochromic anemia being the most prevalent (56%) [14].

Changes in a pregnant woman’s body are immense, and physiological parameters often resemble pathological states, but in fact there is a physiological adjustment of many features. During the gestational period, plasma volume can increase by 40–50% and erythrocyte mass by 18–25%. When there is an increase in plasma volume associated with an expansion in erythrocyte mass, the hemoglobin concentration is altered and hemodilution occurs. Therefore, the diagnosis of anemia during pregnancy can be inaccurate because the levels of hemoglobin in the blood will be lower, especially in the second trimester, but there is a normal physiological adjustment and increase in the third trimester [13, 15]. In this case, the Hb concentration between 100 and 109 g/L for pregnant women will be considered mild, 70–99 g/L as moderate, and below 70 g/L as severe [5].

As anemia during pregnancy is a health problem worldwide, we decided to analyze public data concerning them, with especial attention to low- and middle-income countries (LMICs).

## Methods

We collected epidemiological data of anemia prevalence in pregnant women aged from 15 to 49 years from public dataset available at World Health Organization (https://www.who.int/data/gho/data/indicators/indicator-details/GHO/prevalence-of-anaemia-in-pregnant-women-(-)) and public country income classification at World Data Bank (WDB) (https://datahelpdesk.worldbank.org/knowledgebase/articles/906519). We combined and analyzed those datasets aimed to compare anemia prevalence in pregnant women from 2000 to 2019 at countries with low and low-middle income (LMICs) classified by WDB on 1 July 2021 until 1 July 2022, in approximately 190 countries, with 81 classified as LMICs. World Bank definition of country incomes is: economies are divided among income groups according to 2020 gross national income (GNI) per capita, calculated using the World Bank Atlas method. The groups are: low income, $1045 or less; lower middle income, $1046 to $4095; upper middle income, $4096 to $12,695; and high income, $12,696 or more. Data were analyzed using Excel (Microsoft) and GraphPad Prism version 5.00 for Windows (San Diego, CA, USA, www.graphpad.com, SCR_002798).

## Results

**Fig. 1:**
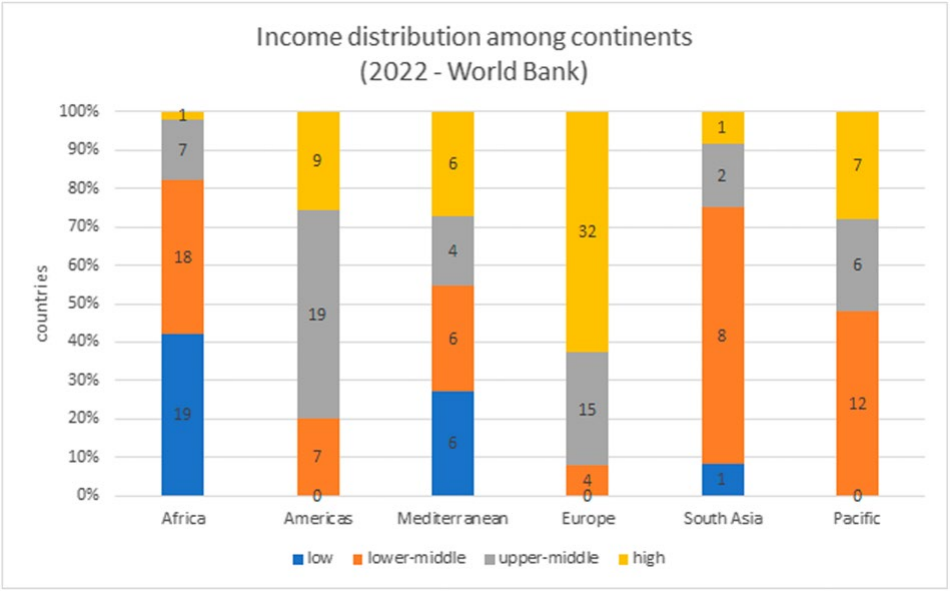
Economies distributions among continents by county incomes from World Bank classification for the fiscal year of 2022. Low-income economies are defined as gross national income (GNI) per capita less or equal as $1045; lower middle-income economies are those with a GNI per capita between $1046 and $4095; upper middle-income economies are those with a GNI per capita between $4096 and $12,695; high-income economies are those with a GNI per capita of $12,696 or more. Numbers in each column represent countries in each income. Data analysis by Excel

The prevalence of anemia varies by geographic region, with the highest occurrence in tropical countries, such as Sub-Saharan Africa, South Asia, the Caribbean, and Oceania. Furthermore, Africa and South Asia have more than 70% of their countries with low- or low-middle income, according to World Bank data (Fig. 1). Overall, 42% of all nations in the world have a population earning less than US$4255 per capita, which classifies them as low- to middle-income, influencing the health of their inhabitants.

**Fig. 2:**
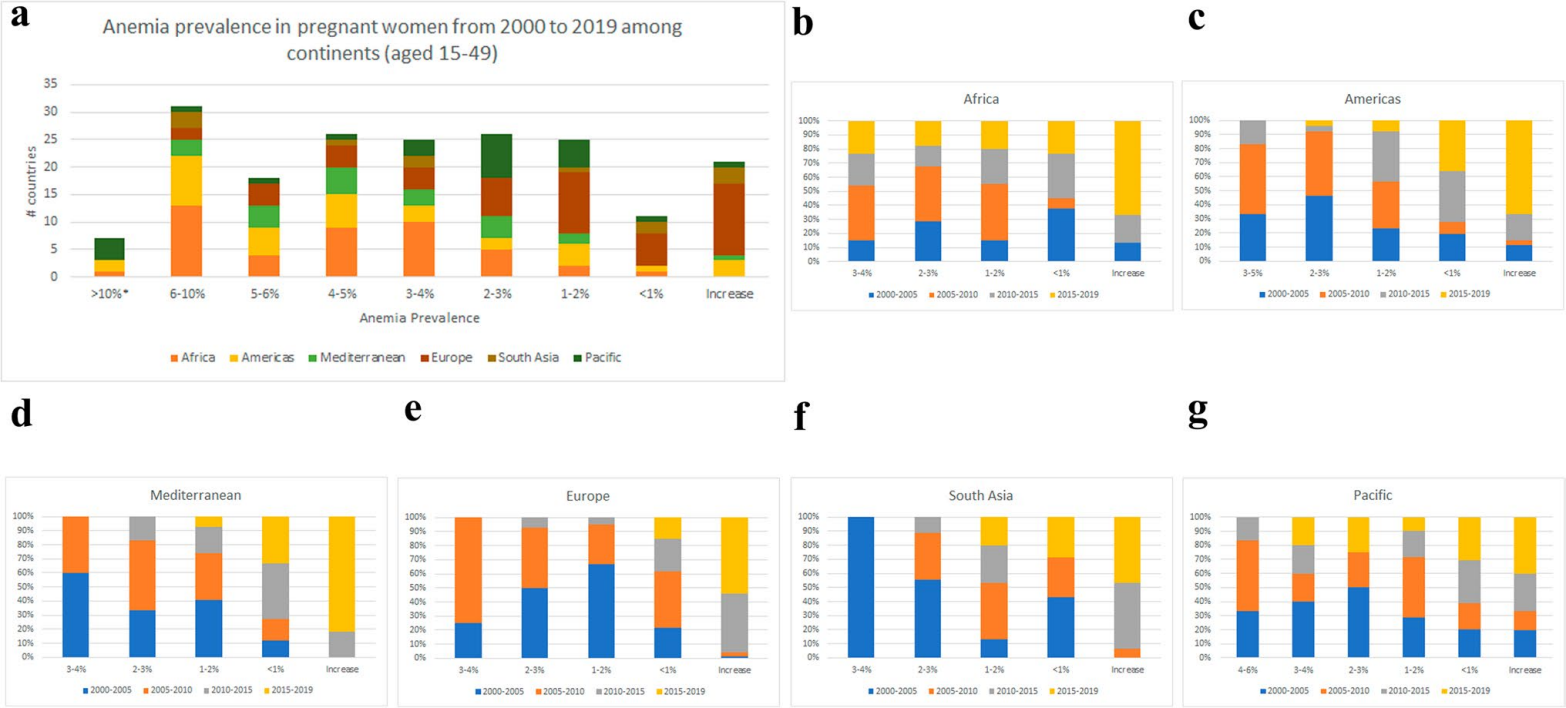
**a** Fluctuation of anemia prevalence in pregnant women among continents in 19 years (2000–2019). **b**–**g** Continent profiles showing anemia prevalence by their tendency in increasing or decreasing prevalence among each 5-year analysis. Abscissa axis shows the percentage of decrease/increase in anemia (%) and the ordinate axis shows how many countries achieved each tendency by 5-year analysis. Data analysis by Excel

**Fig. 3:**
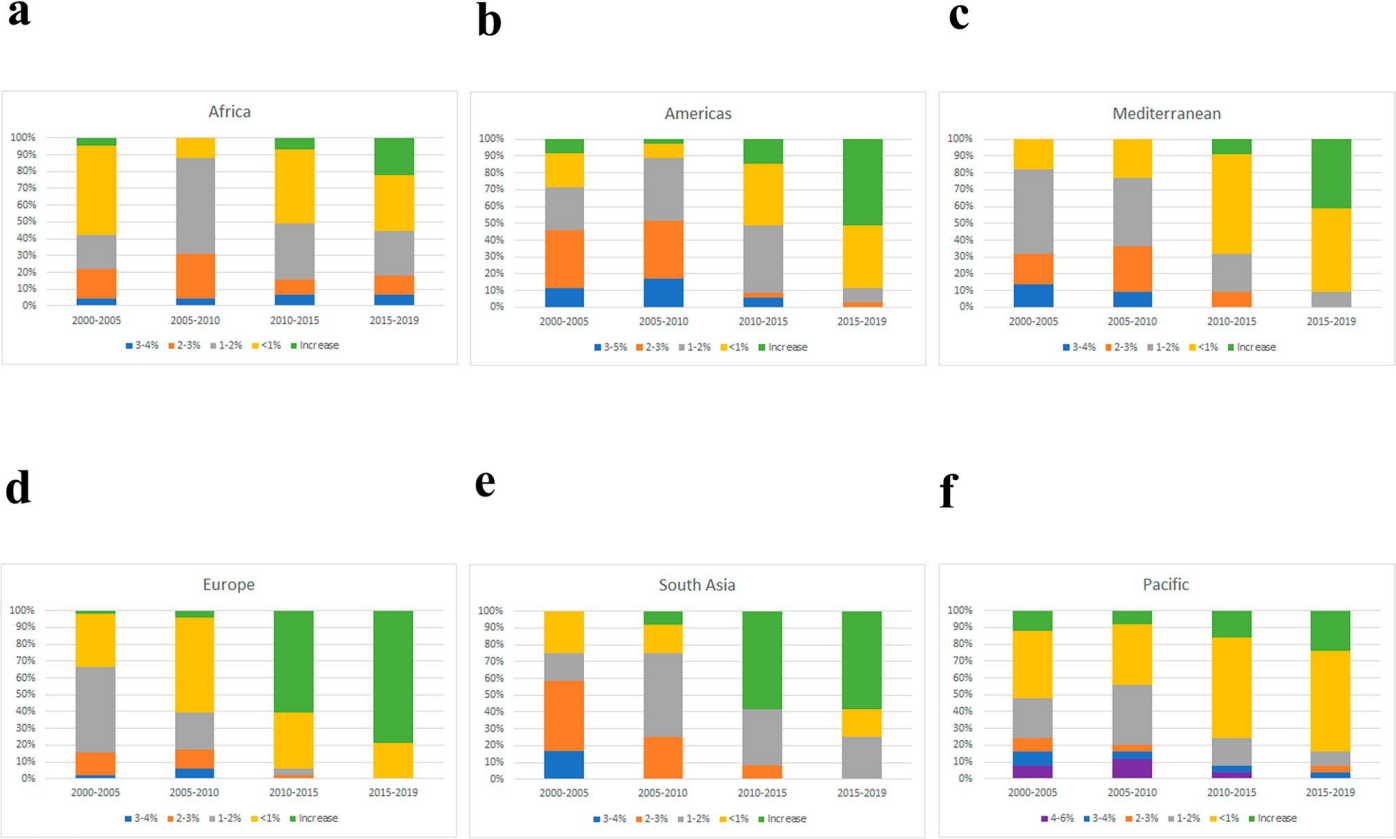
**a**–**f** Prevalence of anemia among continents subdivided from each 5-year period, from 2000 to 2019 in pregnant women. Abscissa axis shows 5-year analysis, and the ordinate axis shows how many countries achieved increased/decreased tendency. Data analysis by Excel

In 19 years, European countries tend to have a lower reduction in anemia among pregnant women, in contrast to Africa and the Americas, with an advanced decrease in prevalence (Fig. 2a). All continents had improved in reducing the prevalence of anemia in pregnant women, especially from to 2005 to 2010 (Fig. 2b–g). South Asia had almost 60% of its countries (7) with at least a 2% drop in prevalence from 2000 to 2005, being the second poorest continent in the world with 75% of its population in low- and lower-middle-income, likewise staying the less numerous. The American continent sustained this drop of just over 2% in around 45–52% of its countries from 2000 to 2010. Additionally, the African continent showed a significant decline in the prevalence of anemia among pregnant women from 2000 to 2010, with 20% (2000–2005) and 35% (2005–2010) of the states showing at least a 2% decrease. This is significant if we consider that this continent has the largest number of states in low- and low-middle income (*n*=37/82%), although much more effort should be made to reach a larger population. Pacific countries had a mild decrease, with less than 20% of its nations revealing more than a 2% reduction in the prevalence of anemia among pregnant women from 2000 to 2019 (Fig. 3a–f).

WHO considers it a significant health problem when anemia prevalence is more than 5% in the population, regarding hemoglobin blood level as a definition parameter [16]. Our evaluation considered a specific cohort population, only pregnant women (from 15 to 49 years old), and our studies confirmed that anemia prevalence fluctuates from 5.2 to 65.7% worldwide, validating it as a public health problem (Table 1). The Americas and Europe have a lower prevalence and are also concentrated in most upper-middle- and high-income countries (*n*=109/57%). The Mediterranean and Pacific have almost equal national distribution from those two incomes and have an intermediate prevalence. Although South Asia is the smallest continent abrogating only twelve nations, it is the second poorest continent, and therefore, displays around 40% of women of reproductive age with anemia. The worst prevalence was in Africa, with almost half of the women with anemia in 2000, but this prevalence dropped by almost 5% until 2019 (Table 1).

**Table 1:** Anemia prevalence in pregnant women from 2000 to 2019 among continents in reproductive age (15–49 years-old)

	2000		2005		2010		2015		2019	
	Mean	Interval	Mean	Interval	Mean	Interval	Mean	Interval	Mean	Interval
Africa	49.2	13.4–64.2	48.0	13–63.4	46.2	11.4–62	45.1	11.3–61.2	44.3	10–61.7
Americas	27.1	6.5–58.2	25.4	6.4–56.4	23.4	5.8–54.6	22.4	5.4–53.9	22.4	5.2–54.4
Mediterranean	37.6	9.7–65.7	35.9	9–65.6	34.3	8.7–65.4	33.6	9.2–65.3	33.3	8.3–65.3
Europe	23.8	6.1–51.3	22.6	6.1–49.2	21.6	6.2–46.9	21.6	6.5–46.4	22.0	6.3–48.2
South Asia	42.5	15.4–57.2	40.6	14–56.3	39.0	12.5–55.2	39.1	10.8–53.9	39.4	9.4–54.8
Pacific	36.8	7.5–61.8	35.6	7.3–61.1	34.1	7.2–60.6	33.1	7.0–59.6	32.6	6.7–58.6
Overall	36.0	6.1–65.7	34.7	6.1–65.6	33.1	5.8–65.4	32.5	5.4–65.3	32.3	5.2–65.3

Data on the prevalence of anemia and/or mean hemoglobin in women of reproductive age, collected between 1995 and 2019 were obtained from 408 population-representative data sources from 124 countries worldwide. A Bayesian hierarchical mixture model was used to estimate hemoglobin distributions and systematically address missing data, non-linear time trends, and representativeness of data sources. Data extracted from WHO (https://www.who.int/data/gho/data/indicators/indicator-details/GHO/prevalence-of-anaemia-in-pregnant-women-(-))

**Fig. 4:**
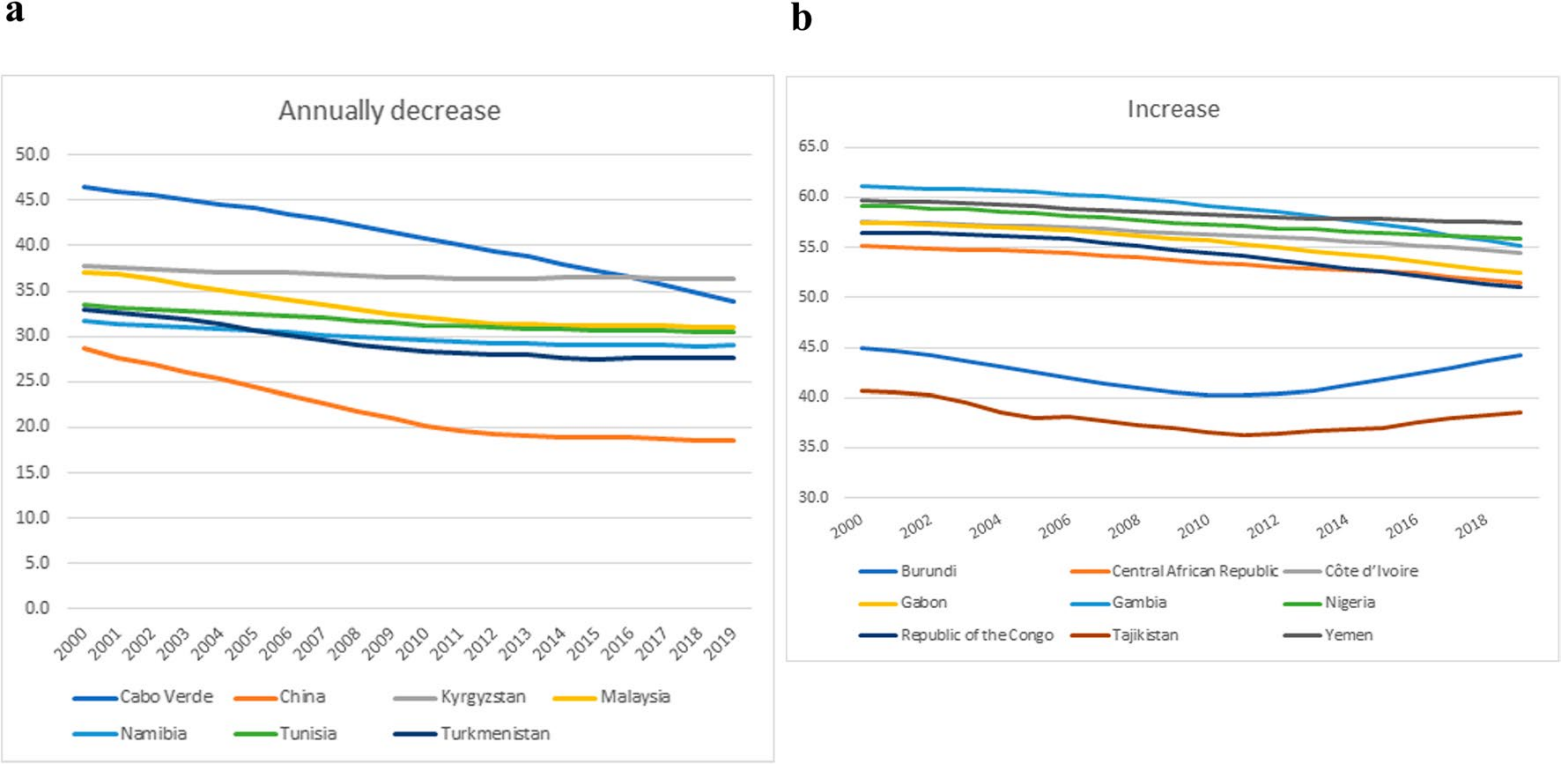
Comparison between GDHx and WHO datasets that drawn attention to fluctuations in anemia among countries. Data analysis by Excel. **a** Seven LMICs experienced annualized decreases in mean anemia prevalence in all of their districts over the 2000–2018 period according to the GHDx dataset. This graph describes the same trend, modeling with the WHO dataset. **b** Nine LMICs showed increases in overall anemia prevalence in the majority of districts according to GHDx. Our analysis using the WHO dataset showed a tendency to decrease in prevalence, at least until 2010, when Gambia and Tajikistan presented annual increases

Kinyoki et al. (2021) [17] also analyzed data from over 3 million women extracted from 218 surveys and correlated them with an extensive geo-positioned dataset from 2000 to 2018, corresponding to 82 LMICs. We also compared our analysis using the WHO dataset with the GHDx dataset; the main conclusions were similar, with 89% (169/190) of overall countries and 97.5% (79/81) of LMICs showing decreases in mean anemia prevalence, whereas they observed 86.6% (71/82). The major difference was the increase in anemia prevalence, in which all nine countries they pointed out at GHDx with the WHO dataset had a mild decrease or a reduction until 2010 with a mild increase until 2019, returning to the same levels observed in 2000 (Fig. 4). We would like to point out nations with a “v” effect that had a huge decrease, with a huge increase like Burundi, Jordan, Albania, Tajikistan, Indonesia, Maldives, and Timor-Leste. Australia, New Zealand, and Singapore had slightly increased in prevalence over the years, although they already had a lower prevalence. In contrast, Cabo Verde, Colombia, Guatemala, China, Philippines, Vanuatu, and Vietnam had a more than 10% decrease in prevalence over 19 years (suppl Fig. 5). These differences could be explained by the fact that the GHDx dataset also contains data from districts, which provides a more detailed overview. Kinyoki et al. also projected that 21 LMICs will maintain high overall anemia in 2030 and 61 nations will have districts with more than 40% anemia prevalence, not achieving the World Health Organization’s Global Nutrition Target. Only three nations are expected to achieve WHO Global Nutrition Target (GNT) by 2030.

## Discussion

Anemia prevalence is a world health problem with almost 90% of the countries with more than 5% of women in reproductive age suffering with the disease. As Africa is the continent with highest incidence of anemia, we looked for data from high-income countries and people from Black ethnic group to comprehend with factor could influence anemia prevalence. A study in the USA from 2003 to 2012 with pregnant Black women revealed that this ethnic group was more likely to have anemia than others [18]. Another study carried out in Italy with low-risk pregnant women without the use of iron supplementation identified that White women had higher concentrations of hemoglobin than Black and Asian pregnant women. The authors also did not observe an association between age, parity, and anemia [19]. In a study by Mohamed et al., the incidence of anemia was higher in African American pregnant women than in Caucasian pregnant women [20]. In a study carried out in Brazil between 2011 and 2012, it was observed that Black pregnant women were more prone to develop arterial hypertension, and no other ethnic group had a correlation with anemia in this study [21]. Iron deficiency is the most cause of worst pregnancy outcomes that which could be worsen when social and educational problems are associated.

The prevalence of anemia appears to decrease with an increase in the educational level of the pregnant women. Women with a high school education or higher are less likely to acquire anemia during pregnancy, probably due to adequate micronutrient intake and health education [22]. Meanwhile, a study carried out in Pakistan revealed that educational level did not show a significant association with anemia in pregnancy, and that lower educational levels were not related to higher risks of anemia in the multivariate regression analysis [23]. Another study in Pakistan with pregnant women in the third trimester indicated that those females with anemia are more prone to adverse pregnancy problems. The main disorders presented are low birth weight, premature birth, gestational hypertension, and preeclampsia, probably due to the iron deficiency associated [24].

Another study conducted in Brazil in native villages from diverse states identified that children born to mothers with anemia were more prone to anemia. In addition, maternal age is associated with the prevalence of childhood anemia, with an increased risk of anemia in children when pregnant women were under 20 years of age [25]. In South Asia, in the 1990s, anemia in pregnant women was around 60–80% [26, 27]. In the same decade, an estimated of 52% of pregnant women from Africa had anemia [28]. In the 2000s, anemia prevalence fluctuates from 42.5 to 39.0% in South Asia and from 49.2 to 44.3% in Africa.

The iron demand during pregnancy increases, which can lead to iron deficiency anemia as iron stores decrease. Anemia is a late manifestation of iron deficiency, and for this reason, the iron concentration in pre-natal period must be sufficient to meet the gestation needs. Pregnant Danish women showed that iron supplementation is essential to mitigate the chances of iron deficiency anemia, as hematological improvements with supplementation were observed [13, 29]. Nonetheless, in Brazil, the Children and Women National Demographic Health Research (PNDS – Pesquisa Nacional de Demografia e Saúde) showed that non-pregnant Black women of reproductive age were more prone to anemia, and in addition, they often started a pregnancy without previous medical follow-up. In addition, women in LMICs are more often prone to nutritional deficiencies in general, which can be aggravated by iron deficiency and anemia during gestation. Poverty is another problem associated with anemia in LMICs, and for this reason, social efforts need to be taken to guarantee minimum food intake every day, as well as food fortification. Once this barrier has trespassed, efforts such as campaigns should be undertaken to motivate women to often get medical follow-ups, get healthy food intake, and take multiple micronutrient supplements when required to decrease anemia, especially before pregnancy to guarantee adequate levels. During pregnancy, such care is really important to avoid complications, especially those associated with children’s development and mother’s outcomes [5, 30, 31]. Another relationship associated with iron deficiency anemia during pregnancy is a high mother mortality rate, probably due to postpartum hemorrhage, which could lead to more dangerous cardiovascular outcomes [31]. At last, multiple efforts from multidisciplinary healthcare professionals such as physicians, gynecologists, obstetricians, nutritionists, and hematologists need to be taken to treat iron deficiency, instead of managing it as supplementation care.

Globally, anemia prevalence among both non-pregnant and pregnant women of reproductive age (WRA) decreased by less than 1% per year (non-pregnant WRA: from 33 to 29%; pregnant WRA: from 43 to 38% between 1995 and 2011, and between 2011 and 2016, it increased from 30 to 33%) [32].

## Conclusions

Anemia prevalence decreased worldwide between 1995 and 2011 and increased from 30 to 33% between 2011 and 2016. Most countries have improved political efforts to encourage women to start prenatal follow-up, implementation of healthcare policies to increase goods with micronutrient fortification, access to fortified food for the low-income population, and an increase in educational politics to reduce worming. The African continent still has more discrepancies compared to other countries around the world, and more aggressive healthcare policies should be implemented to mitigate these disagreements. Genetic and social aspects like educational politics should be considered when managing anemia prevention during pregnancy to mitigate and achieve better results through health education to prevent anemia during pregnancy worldwide.

## Supplementary Information

Below is the link to the electronic supplementary material.

Supp. Fig. 5. Prevalence of anemia profile percontinent (A-F) from to 2000-2019. Source: WHO dataset. Data analysis byPrisma. (PNG 1629 kb)

High resolution image (TIF 11027 kb)

## Data Availability

The raw data that support the findings of this study are available from the WHO repository located at https://www.who.int/data/gho/data/indicators/indicator-details/GHO/prevalence-of-anaemia-in-pregnant-women-(-) and World Bank Country repository located at https://datahelpdesk.worldbank.org/knowledgebase/articles/906519. All the data analyzed by the authors are available upon request.
