# Supplementary figures and images for: Global profile of anemia during pregnancy versus country income overview: 19 years estimative (2000–2019)
Source: Ann Hematol. 2023 May 26;102(8):2025–31. doi: 10.1007/s00277-023-05279-2 (PMC10344983; doi:10.1007/s00277-023-05279-2)

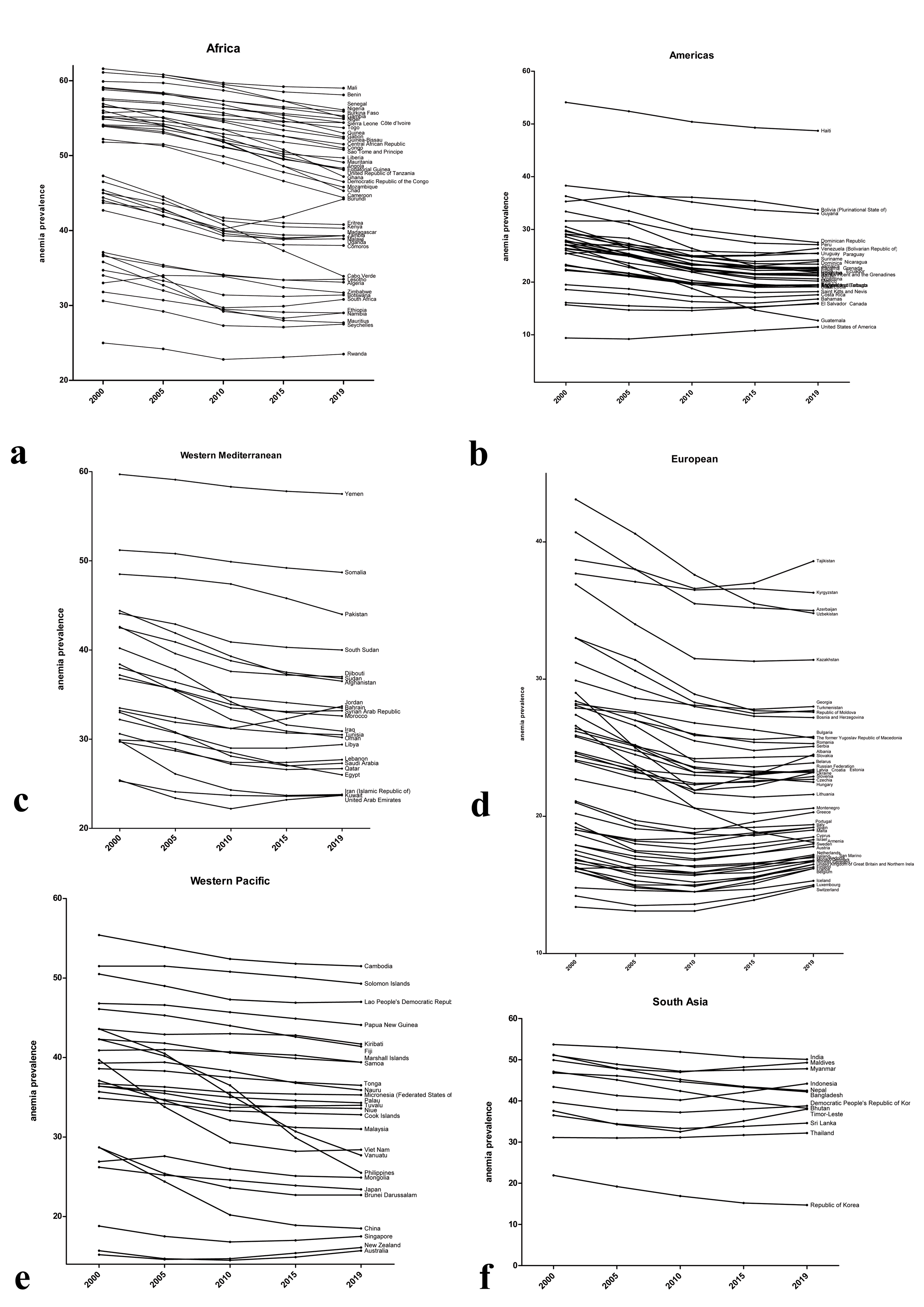

Supplement: Supplementary file 1 — Prevalence of anemia profile percontinent (A-F) from to 2000-2019. Source: WHO dataset. Data analysis byPrisma. (PNG 1629 kb) [file 277_2023_5279_Fig5_ESM.png]

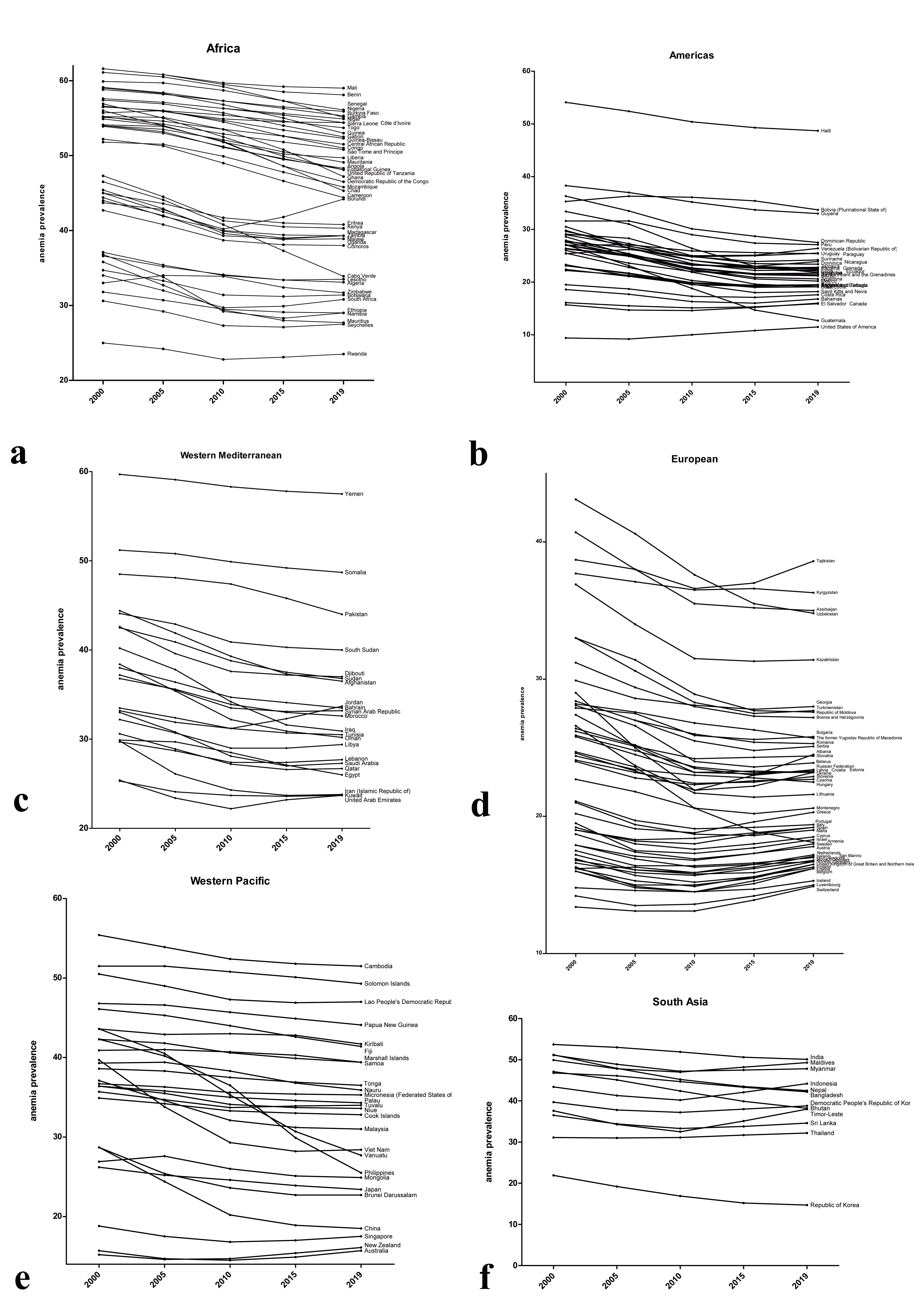

Supplement: Supplementary file 2 — High resolution image (TIF 11027 kb) [file 277_2023_5279_MOESM1_ESM.tif]
